# Novel Antibacterial Modification of Polycarbonate for Increment Prototyping in Medicine

**DOI:** 10.3390/ma14164725

**Published:** 2021-08-21

**Authors:** Tomasz Flak, Ewa Trejnowska, Szymon Skoczyński, Jadwiga Gabor, Beata Swinarew, Klaudia Grzywnowicz, Hubert Okła, Krzysztof Jasik, Arkadiusz Stanula, Grzegorz Brożek, Andrzej S. Swinarew

**Affiliations:** 1 Faculty of Science and Technology, University of Silesia in Katowice, 40-007 Katowice, Poland; tomasz.flak@us.edu.pl (T.F.); jadwiga.gabor@us.edu.pl (J.G.); klaudia.k.kubik@gmail.com (K.G.); hubert.okla@us.edu.pl (H.O.); 2 Department of Cardiac Anesthesia and Intensive Therapy, Silesian Centre for Heart Diseases, Zabrze, Medical University of Silesia, 40-752 Katowice, Poland; ewatrejnowska@gmail.com; 3 Department of Pneumonology, School of Medicine in Katowice, Medical University of Silesia, 40-752 Katowice, Poland; simon.mds@poczta.fm; 4 Lukasiewicz Research Network—Institute for Engineering of Polymer Materials and Dyes, 44-100 Gliwice, Poland; beata.swinarew@impib.lukasiewicz.gov.pl; 5 Department of Pathology, Faculty of Pharmaceutical Sciences in Sosnowiec, Medical University of Silesia in Katowice, 40-752 Katowice, Poland; kjasik3@gmail.com; 6 Institute of Sport Science, The Jerzy Kukuczka Academy of Physical Education, 40-065 Katowice, Poland; a.stanula@awf.katowice.pl; 7 Department of Epidemiology, School of Medicine in Katowice, Medical University of Silesia Katowice, 40-752 Katowice, Poland; gbrozek@sum.edu.pl

**Keywords:** polycarbonate, bacterial biofilm, antibacterial activity, healthcare-related infection

## Abstract

In the era of modern medicine, the number of invasive treatments increases. Artificial devices used in medicine are associated with an increased risk of secondary infections. Bacterial biofilm development observed on the implanted surface is challenging to treat, primarily due to low antibiotics penetration. In our study, the preparation of a new polycarbonate composite, filled with nanosilver, nanosilica and rhodamine B derivative, suitable for three-dimensional printing, is described. Polymer materials with antimicrobial properties are known. However, in most cases, protection is limited to the outer layers only. The newly developed materials are protected in their entire volume. Moreover, the antibacterial properties are retained after multiple high-temperature processing were performed, allowing them to be used in 3D printing. Bacterial population reduction was observed, which gives an assumption for those materials to be clinically tested in the production of various medical devices and for the reduction of morbidity and mortality caused by multidrug-resistant bacteria.

## 1. Introduction

Antibiotic resistance arises as one of the most critical challenges in medicine in the twenty-first century. Due to global antibiotic overuse, in recent decades, a dramatic increase of healthcare-acquired infections caused by multidrug-resistant (MDR) pathogens is being observed. MDR is responsible for rising costs related to infection treatment, but it also leads to significantly increased mortality worldwide. One of the most important reasons for the presence of difficult-to-treat infection is biofilm formation on materials that come into contact with the tissue [1,2].

Biofilm-related infections are responsible for treatment failure and infection recurrence due to the ability of pathogenic microorganisms to survive in the presence of high-antibiotic concentrations. In recent years, there has been an increasing number of difficult-to-treat infections caused by the widespread use of implantable medical devices that predispose to microbial adhesion and patient colonization [3,4].

These chronic tissue- and device-related infections are difficult to treat and associated with a significant risk of recurrence. Typically, in a biofilm-related infection, planktonic bacteria originating from the biofilm spread into the bloodstream. During specified treatment, planktonic bacteria are usually eradicated via the combined action of antimicrobials with cellular and humoral host immune responses. However, a subset of highly tolerant biofilm bacteria frequently survives the antibiotic treatment and can cause infection recurrence [5]. 

The biofilm environment enables bacteria, especially from its deeper layers, to withstand hostile environmental conditions, such as starvation and desiccation, and gives the colony the potential to initiate a whole range of chronic diseases and, therefore, a major cause of persistent nosocomial infections in immune-compromised patients [6]. It is estimated that around 50% of nosocomial infections originate from indwelling devices, such as catheters, cardiac pacemakers, joint prostheses, dentures, prosthetic heart valves and contact lenses [7]. Implantable foreign bodies provide a good surface for bacterial attachment and biofilm formation [6,8].

Therefore, in most cases, removing the colonized device or surgical local tissue excision is the only efficient way to eradicate a biofilm-related infection significantly influencing the outcome and management of infected patients [9]. 

Another important reason for healthcare-associated infections is bacterial transfer by hands from medical furniture. The most commonly used disinfection methods are ethanol, isopropanol, phenol, aldehydes as sterilization and oxidizing agents (e.g., chlorine or other halogens compounds). Each of those solutions should not be used on plastic surfaces [10].

In recent years, a significant increase in bacterial resistance to numerous antibiotics has been observed. Therefore, much effort has been devoted to the development of antibacterial coatings that effectively prevent proliferation or preferably kill bacteria on their surface. Antibiotics can fulfill their role in reducing bacteria population living organisms, but it is tough to use them in the processing of materials. Taking into account probable bacterial resistance, the development of a surface covering with antibiotics would do more harm than good to the environment, because its concentration is already a big problem for many habitats [11].

The aim is to develop coatings with the highest antibacterial activity, while also maintaining high biocompatibility and low cytotoxicity. Currently, the most commonly used elements are silver (Ag) and copper (Cu) [12]. Incorporating powdered Ag or Cu for efficient antibacterial action requires high elemental concentration in the coating, which results in a significant reduction of material stiffness, which, in cases of assured material flexibility, may be used, for instance, for vascular catheters production. However, due to its stiffness reduction and therefore loss of protection ability, it may be inappropriate in terms of coating medical surfaces [13]. Another way of adding antibacterial properties to polymers is a modification with nanoparticles. 

Many methods of modification, such as sol–gel [14], co-precipitation [15], plasma treatment [16], ion implantation [17] or diffusion by gamma radiation [18], are known, but they are changing the only surface of the material. 

Current research shows that metal and metal oxide nanoparticles are impressive antibacterial agents because of their unique properties caused by the size of particles. One of the examples of the practical use of nanoparticles is ZnO- and CuO-coated textiles. Those materials are used for surgical thread production. The results of the antibacterial activity of metal oxide nanoparticles in cultures indicate that the growth of *Staphylococcus aureus* and *Acinetobacter baumannii* was inhibited [19]. The antibacterial properties of nanosilver are commonly known [20,21]. In many cases, nanosilver is used as an active ingredient in composites with nanostructured silica [22]. Until now, nanoparticles were synthesized on the surface of cotton and polyester, but this has not allowed for incorporating them into the substance’s inner structure, which would be useful in medical-device production. In this work, polycarbonates (PC) modification with nanosilver and nanosilica and derivative of rhodamine B showed bacterial population reduction. Additionally, considering their easy and resource-saving properties, there is a possibility to incorporate them into the production of various medical devices.

## 2. Materials and Methods

### 2.1. Nanosilver Modification 

Nanosilver was obtained from Avantor Performance Materials Poland S.A. (Gliwice, Poland) in the form of 30% suspension in ethylene glycol. First, 1 g of suspension was placed in an Erlenmeyer flask equipped with a magnetic stirrer. Then 15 mL of hexane, (Avantor Performance Materials Poland S.A., Gliwice, Poland), was added, and the mixture was stirred for 10 min. After nanosilver precipitate sedimentation, a solution of ethylene glycol in hexane was decanted. The procedure was repeated two times. Residual silver dust was dried at room temperature. Then 4 mL of branched polyol was added. A magnetic stirrer (IKA, Warsaw, Poland) was taken out and switched to the mechanical stirrer (IKA, Warsaw, Poland). The reaction mixture was stirred for 1 h. Afterward, 1.6 mL of pMDI (MINOVA EKOCHEM S.A., Siemianowice Slaskie, Poland) and 0.3 µL of tin(II) 2-ethyl hexanoate OH/NCO catalyst (Nantong Haotai Products&Chemicals Co.,Ltd., Nantong, China) were added. The further reaction was carried out at the reduced pressure of 40 mm Hg. When the crosslinking reaction had taken place, and silver-dust-modified polyurethane was formed, the resulting material was placed in rasper (Chemland, Krakow, Poland), shredded to dust and added to 2000 g of PC. The composition was stirred for 30 min and dried for 24 h, at a temperature of 100 °C. Finally, the mix was blended, and a filament for 3D printing was extruded. The three-zone heating extruder (Brabender Technologie GmbH & Co. KG, Duisburg, Germany) was used, with temperatures starting from 210 to 240 °C, from the first to the last zone. 

### 2.2. Nanosilica Modification

Silica Geduran^®^ Si 60 (40–63 µm) was obtained from Merck KGaA (Darmstadt, Germany). First, 0.3 g of powder was placed in a ball mill reactor (Fritsch, Weinmar, Germany). Then 2 mL of isopropyl alcohol (Avantor Performance Materials Poland S.A., Gliwice, Poland) was added. The reactor was sealed, and the mixture was stirred for a total time of 24 h, at 3600 rpm.

Residual silica dust was dried at room temperature. Then 4 mL of branched polyol was added. The magnetic stirrer was taken out, and we switched to the mechanical stirrer. The reaction mixture was stirred for 1 h. After this time, 1.6 mL of polymeric methylene diphenyl diisocyanate (pMDI) and 0.3 µL of tin(II) 2-ethyl hexanoate OH/NCO catalyst were added. After the crosslinking reaction took place, and silica-dust-modified polyurethane was formed, the resulting material was placed in rasper, shredded to dust and added to 2000 g of PC. The compound was stirred for 30 min and dried for 24 h, at a temperature of 100 °C. Finally, the mix was blended, and a filament for 3D printing was extruded. The three-zone heating extruder was used for this process, with temperatures starting from 210 to 240 °C, from the first to the last zone. 

### 2.3. Rhodamine-B-Derivative Modification

The modifier synthesis started with pouring 20 mL of anhydrous ethanol into the flask and adding a drop of sulfuric acid. Then 2.00 g (7.33 mM) 4-(diphenylamine) benzaldehyde and 0.52 g (3.5 mM) 1,3-indandione were added to the mixture. 

**Figure 1:**
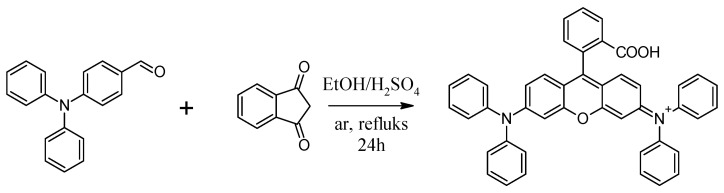
Synthesis of [9-(2-carboxyphenyl)-6-diphenylamine-3-xanthenylidene]-diethylammonium chloride–rhodamine B derivative.

The resulting suspension was rinsed with argon for 15 min, after which the reaction system was equipped with a magnetic dipole(Chemland, Krakow, Poland) and a reflux condenser(Megan S. C., Gliwice, Poland) and heated to reflux under an inert gas atmosphere, stirring vigorously for 24 h. The resulting red reaction mixture was cooled to room temperature. A scheme of the synthesis is presented in Figure 1.

The desired product was isolated by using column chromatography, using SiO_2_ as a stationary phase and a mixture of hexane and methylene chloride as a gradient in the mobile phase. It was then vacuum-dried.

**Figure 2:**
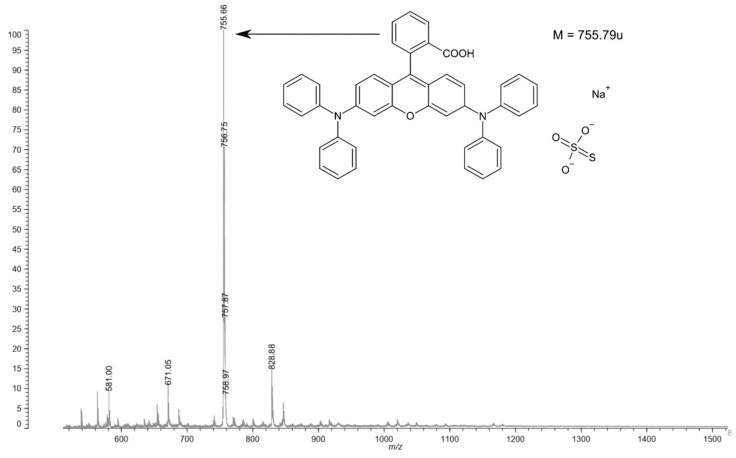
Mass analysis of rhodamine B derivative.

The structure of obtain material was verified (Figure 2) by using a Axima MALDI TOF mass spectrometer(Shimadzu, Kyoto, Japan). Analysis was performed without using a matrix, due to the relatively low mass of the tested compound and good ionization of low-molecular-weight compounds. The mass spectrum shows an ionic association of the obtained chemical with a sulfate ion from the catalytic system and a sodium ion, which probably comes from the glass used during the synthesis.

The filament for 3D FDM printing was obtained in a homogenizer, equipped with a Teflon mechanical stirrer, in which 700 g of Makrolon^®^ 2407 polycarbonate in the form of granules and 0.2 g of dried bacteriostatic rhodamine modifier were placed and mixed until homogeneous polymers surface coverage by the rhodamine derivative. The surface-modified granulate was dried for 24 h, at 100 °C. In the next step, a 2.7 mm diameter string was made by using the temperatures 180, 200, 210 and 225 °C in subsequent heating zones within a four-zone single-screw extruder.

### 2.4. Preparation of the Material for Antibacterial Properties Examination

The plates used for antibacterial properties’ studies were made by using FDM additive technology. They were used to make a polycarbonate-Makrolon^®^ 2407 purchased from Covestro (Leverkusen, Germany), which was then modified by using nano-fillers: a derivative of rhodamine B synthesized according to the procedure described in the patent 231827 “Organic bacteriostatic material”. The plates were made by using the FDM system (Urbicum, Krakow, Poland) with a heated table with a power of 600 W. During the process, the temperature extruder nozzle was maintained at 285 ± 0.25 °C and 165 ± 0.25 °C on the table. The process was carried out in a closed space, without air exchange.

Microbiological studies consisted of checking the effect of PC modification on antiseptic properties. Antibacterial activity was studied according to the microbiological cultivation method; refer to ISO 22196: 2007(E), titled “Plastics Measurement of antibacterial activity on plastics surfaces”. In this study, two reference strains of bacteria, namely *S. aureus* (ATCC 25923) and *E. coli* (ATCC 25922), were used.

The 3D printouts were scanned by using computed microtomography (v|tome|x s, GE Sensing & Inspection Technologies, Phoenix|X-ray, Wunstorf, Germany). First, each sample was placed in a suitable rack and imaged at 100 kV and 150 μA; 1500 projections were recorded with a resolution of 2024 × 2024 pixels. The distance of the sample from the matrix was selected to obtain the maximum image resolution. The parameters determined in this way allowed us to register an image with optimal contrast and a resolution of 20 µm. The acquisition of scans was carried out in 8-bit grayscale, using Datos 2.0 software (GE Sensing & Inspection Technologies, Phoenix|X-ray, Wunstorf, Germany) and VGStudio MAX 2.1 (Volume Graphics, GmbH., Heidelberg, Germany).

Spectrofluorimetric spectra were obtained with the use of FluoroMax-4 spectrofluorimeter(Horiba, Kyoto, Japan) [23].

Chromatographic analyzes were performed by using liquid chromatograph equipped with a light-scattering detector(Shimadzu, Kyoto, Japan) and Shodex GPC-Phenogel column(Showa Denko, Tokyo, Japan) with a grain diameter of 5 µm and a porosity of 500 Å, 300 mm long and 7.8 mm wide. Tetrahydrofuran was used as the eluent, and the flow rate was 1 cm^3^/min. The volume of a single injection was set to 100 μL. The process was carried out at a temperature of 40 °C. Calibration was performed by using Aldrich polystyrene standards [24].

Infrared spectroscopy measurements were performed by using IR Tracer-100(Shimadzu, Kyoto, Japan) equipped with ATR accessory (attenuated total reflectance, ATR) [23].

## 3. Results

**Figure 3:**
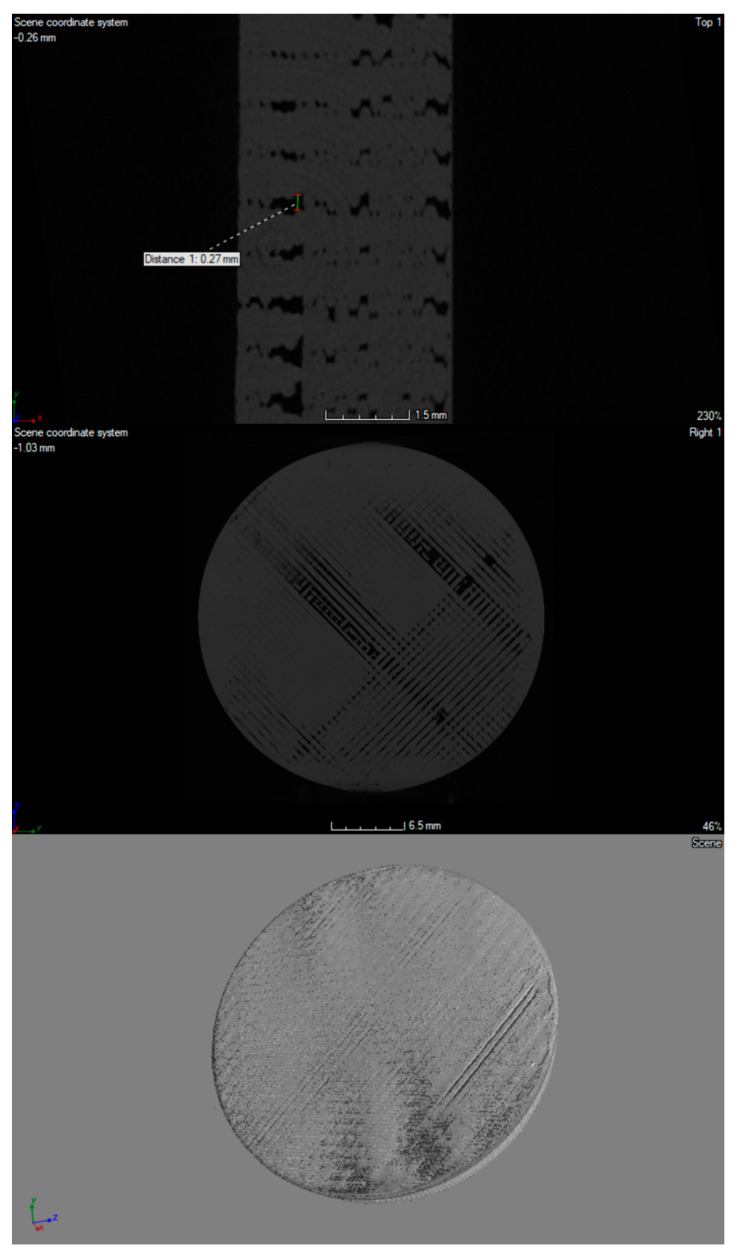
Micro CT imaging of 3D-printed porous standard plate modified with nanosilver.

**Figure 4:**
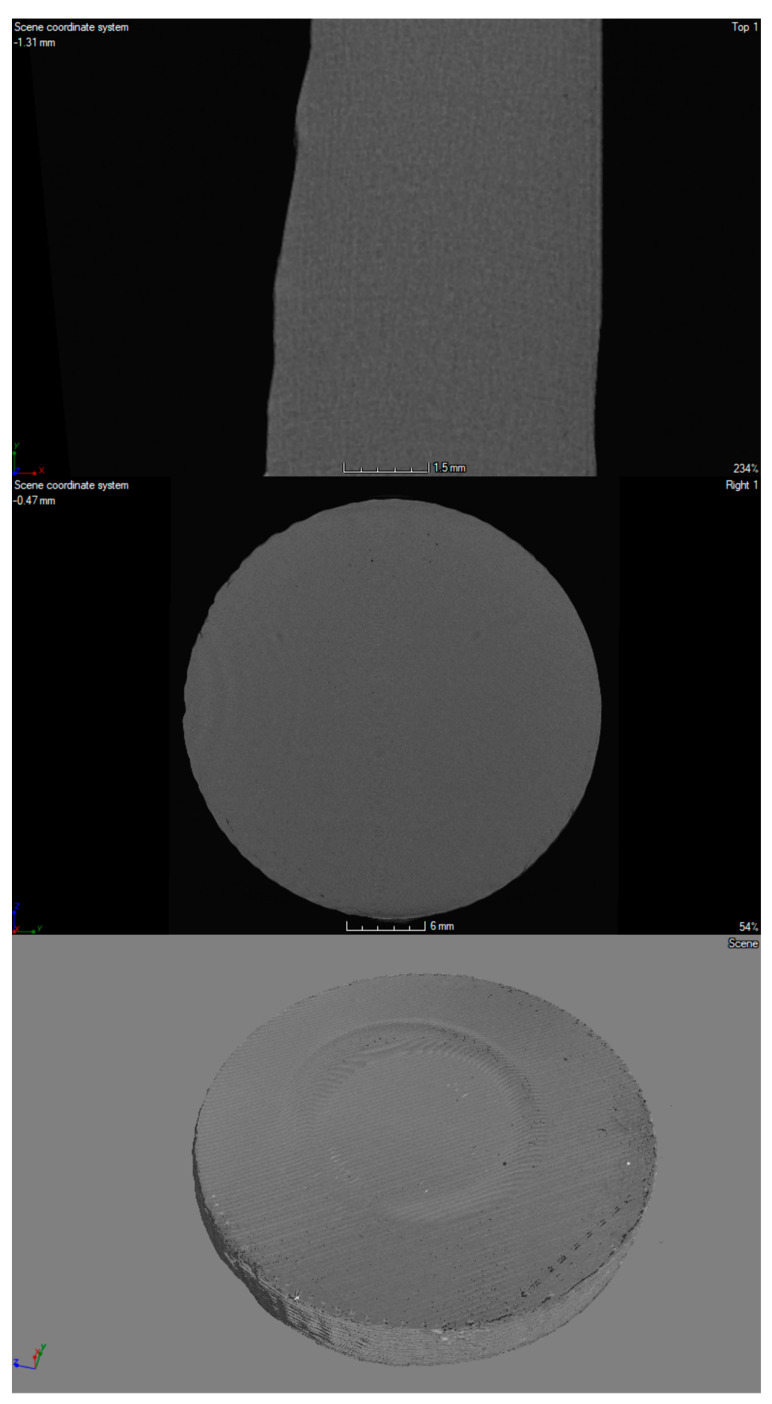
Micro CT imaging of 3D-printed porous standard plate modified with nanosilica.

**Figure 5:**
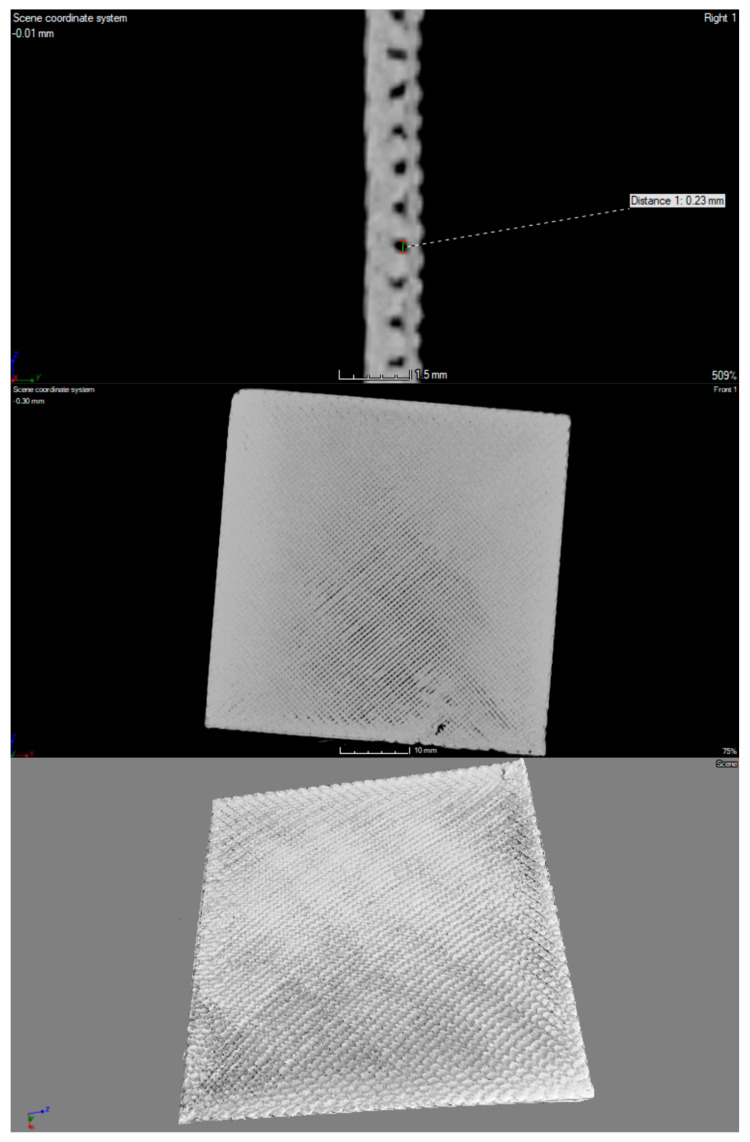
Micro CT imaging of 3D printed porous standard plate modified with rhodamine B derivative.

A high-resolution X-ray scanner GE Phoenix v | tome | x was used for visualization and spatial imaging of plates in their entire volume (Figure 3, Figure 4 and Figure 5). In the structure of the plates made of material with the addition of nanosilver and rhodamine, the print path was visible, which was within the range of 0.23–0.27 mm. In the case of a material modified with nanosilica, the print path was invisible; it may be related to the nature and properties of this material. All of the tested prints showed a homogeneous microstructure with no signs of material agglomeration. Moreover, no hypodense areas were observed in the microtomographic examination.

Cytotoxic properties studies used the primary line of adipose-derived stem cells (ADSCs) purchased from Lonza. For the evaluation of cytotoxicity, the MTT tetrazolium salt (methyl-thiazolyl-diphenyl-tetrazole, 3-(4,5-dimethyl-2-thiazolyl)-2,5-diphenyl-2H-tetrazolium bromide) (purchased from Sigma-Aldrich) was used.

In order to determine the impact of multiple processing procedures of the modified material on its properties and structure, both during the preparation process and final processing and printing, its tests were performed by using spectrofluorimeter, infrared spectroscopy (FTIR) and gel-permeation chromatography (GPC).

**Figure 6:**
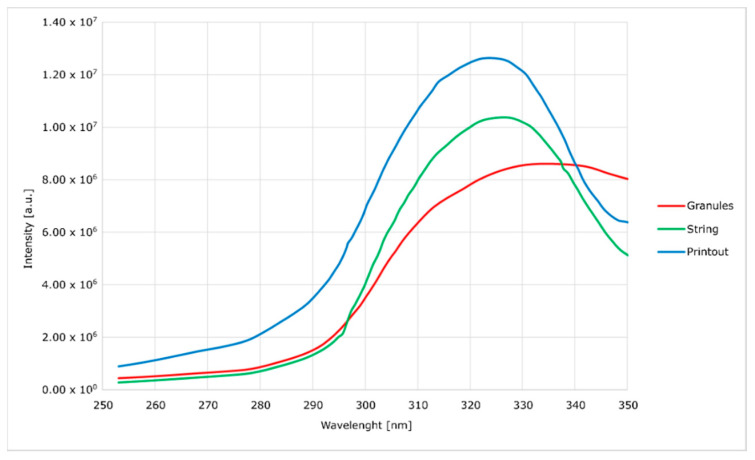
Excitation spectra of nanosilica-modified PC.

**Figure 7:**
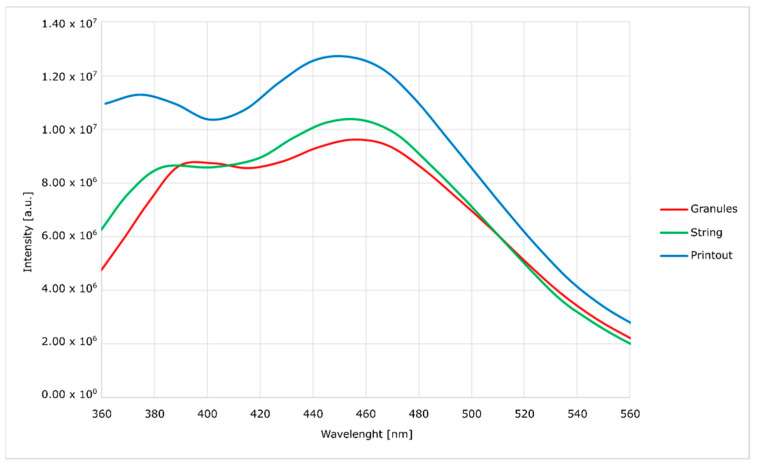
Emission spectra of nanosilica-modified PC.

**Figure 8:**
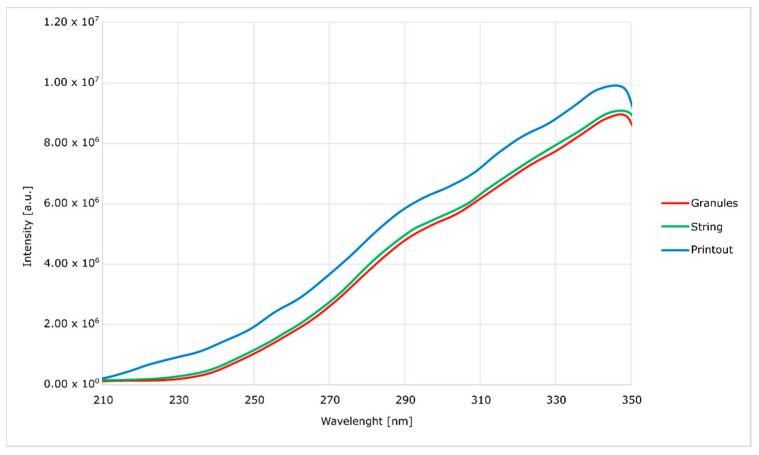
Excitation spectra of nanosilver-modified PC.

**Figure 9:**
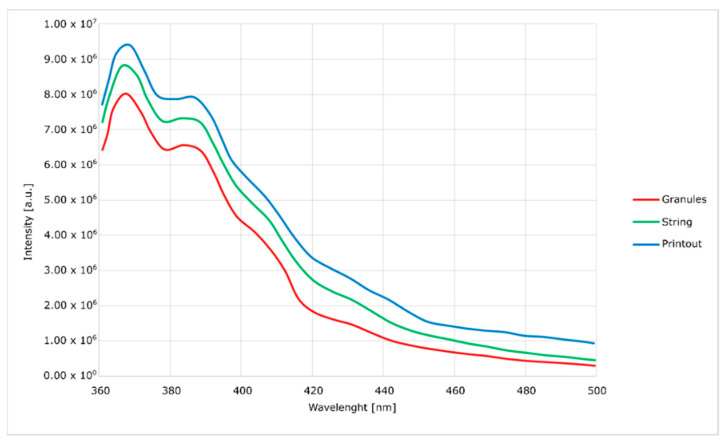
Emission spectra of nanosilver-modified PC.

Spectrofluorimetric measurements (Figure 6, Figure 7, Figure 8 and Figure 9) showed that it is possible to determine the presence of specific modifications in prepared samples of PC. It is also important that the experiment confirmed that the use of polymers matrix could reduce agglomeration of particles. An increase can see it in the intensity of graph values if we compare modified granules and final printouts. In our opinion, these differences are caused by a more homogenous distribution of modifier in the total volume of the matrix than only on the surface. Moreover, printing from modified strings changes the distribution one more time, creating a more unified material.

The emission and excitation tests, using a spectrofluorimeter, showed significant differences between the modified granulate, filament and the printout. With each stage of high-temperature processing (i.e., extrusion and FDM printing), the intensity of the optical response increases. It proves the lack of degradation of the active material and better homogenization of the entire composite. When the concentration of optically active substances is high, low-energy agglomerates may form, and this reduces the emission of radiation. The opposite tendency with the subsequent mixing of the plasticized material with the modifier allows us to assume that the mentioned agglomerates are broken and separated.

The emission curves for the modified material, with two peaks in the range visible on the spectra, are characteristic of polycarbonate. In the literature, it was noted that the occurrence of shifts depends on the method of material preparation. In the case of this work, the curves generally show no hypsochromic and bathochromic shifts. The only exception is the curve corresponding to the emission of the silica-containing granulate. This effect is attributed to the high concentration of silica in the surface layer of the granules without complete homogenization. This theory is confirmed by a significant blurring of the excitation band, which does not occur in other cases. This effect disappears after the additive penetrates the polymer in the extrusion process. Additionally, in the presented spectra (Figure 6, Figure 7, Figure 8 and Figure 9), an increase in the intensity of the bands can be seen. The reason for this effect is the better contact of the radiation with the polymer. This trend also shows (as does the infrared spectrum) no degradation of the polymer. The strong optical activity of the rhodamine B derivative does not represent an analogous description of polycarbonate-based on the fluorimetric spectrum.

**Figure 10:**
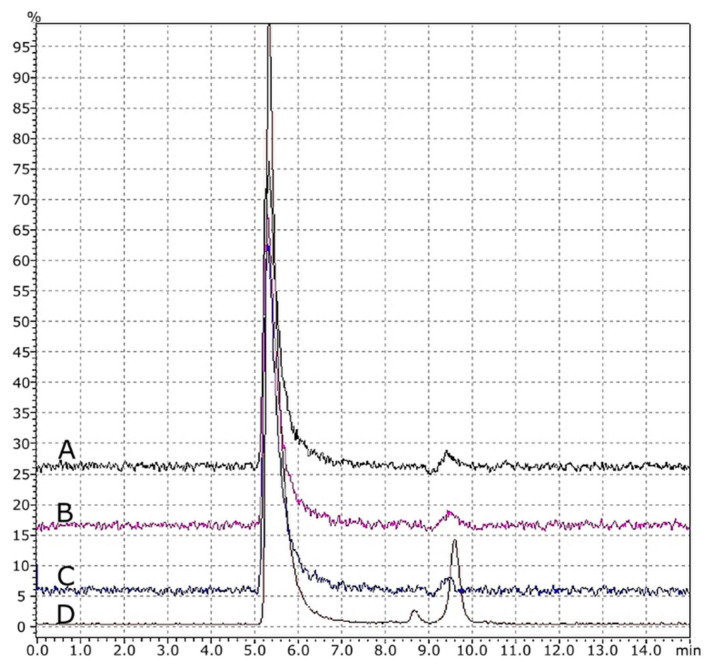
GPC chromatograms: A = printout, B = string, C = modified granules and D = pure PC granules.

**Figure 11:**
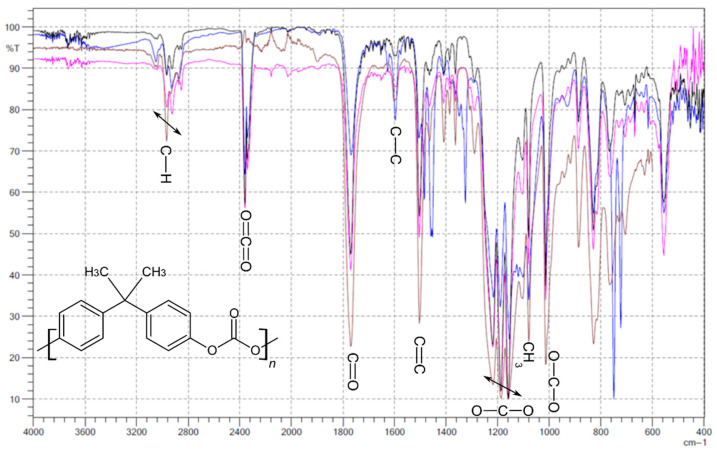
FTIR spectra: black line = printout, pink line = string, blue line = modified granules and brown line = pure PC granules.

Additionally, based on obtained results from GPC (Figure 10) and FTIR analysis (Figure 11), it was found that thermal treatment on the material did not have any negative effects. There were no degradation products found. There were no degradation products found. All of the characteristic signals for polycarbonate were identified, as shown in Figure 11. No changes were seen to indicate thermal degradation of any polymer forms (granules, strings and printouts). The presented selected results are representative for all groups of the conducted analyzes (nanosilver, nanosilica and rhodamine B derivative).

Evaluation of the antibacterial activity of the modified polyester surface was made in accordance with ISO 22196: 2011 (E), “Plastics Measurement of antibacterial activity on plastics surfaces”. The reference strains, namely *Staphylococcus aureus* (DSM 346) and *Escherichia coli* (ATCC 25922), were used as the reference material. The inoculum volume at 6 × 10^5^ cells/mL was 0.4 mL. All culture media and solutions were made according to the standard, sample size 50 mm × 50 mm, cover film PP film 40 mm × 40 mm and 0.05 mm thickness; 10 mL of SCDLP broth was used as the neutralizer.

R = (U_t_ − U_0_) − (A_t_ − U_0_) = U_t_ − A_t_ (1)

where U_0_ is the average logarithm of the number of live bacteria, number of cells/cm^2^, recovered from untreated samples after culturing; U_t_ is the average logarithm of the number of live bacteria, number of cells/cm^2^, recovered from untreated samples after 24 h; and A_t_ is the average of the logarithm of the decimal number of live bacteria, the number of cells/cm^2^, recovered from samples treated after 24 h.

The R factor allows us to quantify the impact of the tested material on reference bacteria strains. The conducted studies of antibacterial properties have shown that the used modifiers (nanosilica, nanosilver and rhodamine B) have properties that reduce the growth of *E. coli* and *S. aureus* (Table 1).

The antimicrobial properties are based on the following classifications:

- R ≤ 0.5 no antimicrobial activity (68.4% reduction of microbial growth);
- 0.5 < R ≤ 1 slight antimicrobial activity (68.4% to 90% reduction of microbial growth);
- 1 < R ≤ 2 medium antimicrobial activity (90% to 99% reduction of microbial growth);
- 2 < R ≤ 3 good antimicrobial activity (99% to 99.9% reduction of microbial growth);
- R > 3 very good antimicrobial activity (>99.9% reduction of microbial growth) [25].

In the case of the material with nanosilica and rhodamine B, a greater reduction in growth of the bacteria was observed than in the case of the material with nanosilver.

Cell viability was tested as a percentage of viable cells in the individual test groups in relation to control cells. According to the guidelines, viability above 70% indicates no cytotoxic effect. The tests showed that none of the tested samples showed any cytotoxic activity against the murine fibroblasts of the L-929 line.

## 4. Discussion

According to our early results, it may be assumed that the unique antibacterial properties of developed materials give an assumption of fast clinical tests and early introduction into clinical medicine. The advantages of presented materials may be used to produce various implantable devices, both long-lasting, such as cardiac pacemakers or surgical threads, and used for an intermediate time, such as airway or vascular catheters used in intensive care worldwide. Another field for possible implementation is to use it to cover medical surfaces, which play an important role in infection transmission. This innovation, if tested in clinical trials it, will probably prove that the use of these substances will decrease the number of patients with bacterial biofilm development, and therefore antibiotic consumption; decrease the need for surgical re-intervention, as well as antibiotics consumption; and therefore decrease (multi/extensive/pan drug-resistant) MDR/XDR/PDR-pathogens. Therefore, the materials fulfill the definition of preventive medicine, which is accepted as a resource and found to be life-saving.

It is estimated that increasing antimicrobial resistance (AMR) without effective preventive actions may be responsible for approximately 10 million deaths, and their treatment may cost as much as 100 trillion US dollars per year by 2050. It is important to underline that, in contrast to some other health issues, AMR is a global burden that is present in all economies [1,2]. On the population level, the treatment of bacterial infections is challenging: firstly, because of common antibiotic overuse and a significant secondary decrease of still useful antibiotics (susceptible bacteria); and secondly, to the slow rate of new drug development. In recent years, colistin was reserved as a last-line antibiotic that was frequently administered as a last-line treatment of Gram-negative multiresistant infections. However, a new gene (mcr-1) discovered in 2015 was found to enable bacteria to be highly resistant to polymyxins (such as colistin). This gene is susceptible to horizontal transfer, which gives the potential to be spread between different bacterial populations, facilitating the bacterial transformation from “extensive drug resistance” to “pan-drug resistance [5].

Typically, in a biofilm-related infection, planktonic bacteria originating from the biofilm spread into the bloodstream, causing generalized infections such as sepsis, or spread around the implantable device, causing local infections, such as abscesses in chronic bacteremia.

The additional features of our novel material give the possibility to implement it both for the production of flexible devices, such as an intravascular catheter and artificial airways, such as tracheal tubing; or tubing of various devices which are in contact with blood flow, such as Extracorporeal Membrane Oxygenation (ECMO) or dialysis machines. Moreover, it also functions as an outer layer of stiff devices, such as implantable cardiac devices. The implementation of our material is promising, especially in a hospital setting, as it was reported that a substantial number of healthcare-acquired infections are preventable [26]. It is especially important in the case of IC medicine, because it may induce immunosuppression and promote the development of further hospital-acquired infections [27].

This is especially important, because the use of strong-acting germicidal antibiotics, such as imipenem and colistin, can only reduce the volume of biofilm, but cannot eliminate it entirely. Due to antibiotic toxicity and numerous side effects, it is not possible to reach the minimal concentration of antibiotic in vivo in the biofilm structure. The higher values of MIC and minimal bactericidal concentration (MBC) for the intra biofilm bacterial cells have therefore made the antibiotic treatment less effective [17,19]. Biofilms protect the invading bacteria against the host immune system via impaired activation of phagocytes and complement system and also increase their resistance against the conventional antibiotics by around 1000-fold; however, this mechanism is effective, especially in conductive airways [6,28].

It has to be underlined that all implantable devices are prone to mechanical destruction. In actually used devices with antibacterial cover only, there is even a more significant chance for biofilm development.

The major innovation of the method developed by us can be applied to add antibacterial properties to the interior of polymer to provide reasonable protection even in the case of the damaging exterior layer.

Those properties depend on size and morphology to overcome the cohesion forces of a particular particle. There are a few main mechanisms of bacterial-growth inhibition. The antiseptic properties of nanoparticles are associated with cytoplasmic-membrane and cell-wall destruction, homeostasis disorder, DNA destruction and inactivation of proteins, e.g., bacterial enzymes. Multidirectional interactions of nanoparticles with cell organelles and biochemical pathways reduce the risk of drug resistance in bacteria. However, the antibiotic-resistance phenomenon is the reason for the ineffectiveness of these substances [29].

The first most intuitive implication is the use of our material for central-line production. Up till now, different substances were tested for central-line coating; however, currently, the results are negative in the case of chlorhexidine [30], and in the case of silver-coated devices, a 32% likelihood of central-line-associated bloodstream infection was observed [31].

The second possible clinical implementation would be for urinary-catheter production. In an outpatient setting, there is a significant number of patients with chronic urinary bladder catheterization.

Those patients are prone to numerous complications requiring emergency-department visits. Local and systemic infections are the most common and potentially life-threatening complications, which may range from a simple urinary bladder infection to MDR urosepsis [32].

The approach we proposed to prevent or decrease the likelihood of catheter-associated urinary-tract infections is a burning clinical problem that has been addressed already by an attempt to produce “antibacterial catheters”. One of the approaches was presented in a study by Saini H et al., who demonstrated the effectiveness of azithromycin–ciprofloxacin impregnated urinary catheters in preventing catheter-associated urinary tract infections in a long-term in vivo a murine model [33].

Although promising, according to our best knowledge, this has not yet been tested on a human model. Moreover, the antibiotic coating creates a possibility to induce MDR Pseudomonas Aeruginosa clones, which is especially risky for ciprofloxacin [34,35], which is commonly used in outpatient setting.

Production of bacteriostatic cardiological implantable devices is of great importance, as cardiac device infective endocarditis is a complication that may be present in over 5% of implanted patients, frequently requires cardiosurgical removal and is associated with significant mortality [36]. Implantable cardiac devices are used globally. On the other hand, the number of patients who undergo surgical treatment is much higher, and surgical site infections are accepted as one of the most common surgical treatment complications. Antibiotic prophylaxis is frequently introduced to decrease this risk. Unfortunately, it does not guarantee freedom from infections. It has to be underlined that the surgical-site infections (SSIs) were reported to be present in up to 10% of patients with a colonized thread, whereas there was no SSI in patients without thread colonization [37]; therefore, introduction into surgical thread production should also be considered.

Airway cannulation represents another possible field of medical implementation, because it was found that long-term tracheostomy is frequently associated with an increase in multidrug-resistant bacilli colonization [38]. There are already suggestions to test silver-coated tracheotomy tubes to check whether their usage may decrease the risk of ventilatory acquired pneumonia [39], which facilitates our suggestion to use our material for airway tubes production. Therefore, another possible implementation may be for the production of tracheostomy tubes, especially for patients which will be chronically tracheotomized, or intubation tubes dedicated for longer treatment, such as ICU implementation.

We are fully aware of our primary study limitation, which is namely caused by the performance of in vitro tests only; however, looking at our early results, it may be expected that the product will be easily implemented into the production of various medical devices. However, it has to be tested in prospective clinical studies.

Therefore, our results should be easily used by manufacturers, and the antibacterial activity should be compared with other currently available techniques.

## 5. Conclusions

To conclude, all presented materials are entirely composed of an antibacterial structure, which decreases the likelihood of biofilm development to a much more considerable extent than the currently used materials. Therefore, they are suitable for clinical testing. There is an excellent likelihood that they may be widely used to produce numerous medical devices and cover medical surfaces.

## 6. Patents

Part of the results of the work is protected by intellectual property rights. Patent obtained: “Organic bacteriostatic material” PL231827 (B1)—2019-04-30.

## Figures and Tables

**Table 1:** Evaluation of the antibacterial activity of the modified polyester surface.

Modifier	U_o_	U_t_	A_t_	R
Nanosilica	4.22	4.91	3.84	1.10
Nanosilver	4.44	5.58	4.82	0.76
Rhodamine B Derivative	4.21	4.91	3.46	1.45

## Data Availability

The data underlying this article will be shared on reasonable request from the corresponding author.

## References

[1] WHO Antimicrobial resistance (2014). Global report on surveillance. World Health. Org..

[2] O’Neill J. (2016). Tackling Drug-Resistant Infections Globally: Final Report and Recommendations: The Review on Antimicrobial Resistance. https://amr-review.org/sites/default/files/160518_Final%20paper_with%20cover.pdf.

[3] Donlan R.M., Costerton J.W. (2002). Biofilms: Survival mechanisms of clinically relevant microorganisms. Clin. Microbiol. Rev..

[4] Lewis K. (2007). Persister cells, dormancy and infectious disease. Nat. Rev. Microbiol..

[5] Viganor L., Howe O., McCarron P., McCann M., Devereux M. (2016). The Antibacterial Activity of Metal Complexes Containing 1,10-phenanthroline: Potential as Alternative Therapeutics in the Era of Antibiotic Resistance. Curr. Top. Med. Chem..

[6] Roy R., Tiwari M., Donelli G., Tiwari V. (2018). Strategies for combating bacterial biofilms: A focus on anti-biofilm agents and their mechanisms of action. Virulence.

[7] Wu H., Moser C., Wang H.Z., Høiby N., Song Z.J. (2015). Strategies for combating bacterial biofilm infections. Int. J. Oral Sci..

[8] Donelli G., Francolini I. (2001). Efficacy of antiadhesive, antibiotic and antiseptic coatings in preventing catheter-related infections: Review. J. Chemother..

[9] Lebeaux D., Ghigo J.M., Beloin C. (2014). Biofilm-Related Infections: Bridging the Gap between Clinical Management and Fundamental Aspects of Recalcitrance toward Antibiotics. Microbiol. Mol. Biol. Rev..

[10] Alariqi S.A.S., Mutair A.A., Singh R.P. (2016). Effect of Different Sterilization Methods on Biodegradation of Biomedical Polypropylene. J. Environ. Anal. Toxicol..

[11] Mojica E., Aga D. (2011). Antibiotics Pollution in Soil and Water: Potential Ecological and Human Health Issues. Encyclopedia of Environmental Health.

[12] Musil J. (2017). Flexible antibacterial coatings. Molecules.

[13] Musil J., Blažek J., Fajfrlík K., Čerstvý R., Prokšová Š. (2013). Antibacterial Cr-Cu-O films prepared by reactive magnetron sputtering. Appl. Surf. Sci..

[14] Gollwitzer H., Haenle M., Mittelmeier W., Heidenau F., Harrasser N. (2018). A biocompatible sol–gel derived titania coating for medical implants with antibacterial modification by copper integration. AMB Express..

[15] Alam M., Ansari A.A., Shaik M.R., Alandis N.M. (2013). Optical and electrical conducting properties of Polyaniline/Tin oxide nanocomposite. Arab. J. Chem..

[16] Mackova A., Svorcik V., Stryhal Z., Pavlik J. RBS and AFM study of Ag and Au diffusion into PET foils influenced by plasma treatment. Proceedings of the 11th European Conference on Applications of Surface and Interface Analysis.

[17] Prakash J., Tripathi A., Rigato V., Pivin J.C., Tripathi J., Chae K.H., Gautam S., Kumar P., Asokan K., Avasthi D.K. (2011). Synthesis of Au nanoparticles at the surface and embedded in carbonaceous matrix by 150 keV Ar ion irradiation. J. Phys. D. Appl. Phys..

[18] Akhavan A., Sheikh N., Khoylou F., Naimian F., Ataeivarjovi E. (2014). Synthesis of antimicrobial silver/hydroxyapatite nanocomposite by gamma irradiation. Radiat. Phys. Chem..

[19] Perelshtein I., Lipovsky A., Perkas N., Tzanov T., Arguirova M., Leseva M., Gedanken A. (2015). Making the hospital a safer place by sonochemical coating of all its textiles with antibacterial nanoparticles. Ultrason. Sonochem..

[20] Guo L., Yuan W., Lu Z., Ming Li C. (2013). Polymer/nanosilver composite coatings for antibacterial applications. Colloids Surf. A.

[21] Li B., Moriarty T.F., Webster T., Xing M. (2020). Racing for the Surface: Pathogenesis of Implant Infection and Advanced Antimicrobial Strategies.

[22] Tang J., Tang D., Su B., Li Q., Qiu B., Chen G. (2011). Nanosilver-penetrated polyion graphene complex membrane for mediator-free amperometric immunoassay of alpha-fetoprotein using nanosilver-coated silica nanoparticles. Electrochim. Acta..

[23] Larosa C., Patra N., Salerno M., Mikac L., Meri R.M., Ivanda M. (2017). Preparation and characterization of polycarbonate/multiwalled carbon nanotube nanocomposites. Beilstein J. Nanotechnol..

[24] Jenke D. (2003). Chromatographic Methods Used to Identify and Quantify Organic Polymer Additives. J. Liq. Chromatogr. Relat. Technol..

[25] Scuri S., Petrelli F., Grappasonni I., Idemudia L., Marchetti F., Di Nicola C. (2019). Evaluation of the antimicrobial activity of novel composite plastics containing two silver (I) additives, acyl pyrazolonate and acyl pyrazolone. Acta Biomed..

[26] Schreiber P.W., Sax H., Wolfensberger A., Clack L., Kuster S.P. (2018). The preventable proportion of healthcare-associated infections 2005-2016: Systematic review and meta-analysis. Infect. Control Hosp. Epidemiol..

[27] Van Vught L.A.V., Klouwenberg P.M.C.K., Spitoni C., Scicluna B.P., Wiewel M.A., Horn J., Schultz M.J., Nürnberg P., Bonten M.J.M., Cremer O.L. (2016). Incidence, risk factors, and attributable mortality of secondary infections in the intensive care unit after admission for sepsis. JAMA-J. Am. Med. Assoc..

[28] Bjarnsholt T., Jensen P.Ø., Fiandaca M.J., Pedersen J., Hansen C.R., Andersen C.B., Pressler T., Givskov M., Høiby N. (2009). Pseudomonas aeruginosa biofilms in the respiratory tract of cystic fibrosis patients. Pediatr. Pulmonol..

[29] Pokrowiecki R. (2013). Evaluation of biocidal properties of silver nanoparticles against cariogenic bacteria. Med. Dosw. Mikrobiol.

[30] Storey S., Brown J., Foley A., Newkirk E., Powers J., Barger J., Paige K. (2016). A comparative evaluation of antimicrobial coated versus nonantimicrobial coated peripherally inserted central catheters on associated outcomes: A randomized controlled trial. Am. J. Infect. Control.

[31] Jacob J.T., Tejedor S.C., Reyes M.D., Lu X., Easley K.A., Aurand W.L., Steinberg J.P. (2015). Comparison of a silver-coated needleless connector and a standard needleless connector for the prevention of central line-associated bloodstream infections. Infect. Control Hosp. Epidemiol..

[32] Ansell T., Harari D. (2017). Urinary catheter-related visits to the emergency department and implications for community services. Br. J. Nurs..

[33] Saini H., Vadekeetil A., Chhibber S., Harjai K. (2017). Azithromycin-ciprofloxacin-impregnated urinary catheters avert bacterial colonization, biofilm formation, and inflammation in a murine model of foreign-body-associated urinary tract infections caused by Pseudomonas aeruginosa. Antimicrob. Agents Chemother..

[34] Peng J., Cao J., Ng F.M., Hill J. (2017). Pseudomonas aeruginosa develops Ciprofloxacin resistance from low to high level with distinctive proteome changes. J. Proteomics.

[35] Serra C., Bouharkat B., Touil-Meddah A.T., Guénin S., Mullié C. (2019). MexXY multidrug efflux system is more frequently overexpressed in ciprofloxacin resistant French clinical isolates compared to hospital environment ones. Front. Microbiol..

[36] Athan E., Chu V.H., Tattevin P., Selton-Suty C., Jones P., Naber C., Miró J.M., Ninot S., Fernández-Hidalgo N., Durante-Mangoni E. (2012). Clinical characteristics and outcome of infective endocarditis involving implantable cardiac devices. JAMA J. Am. Med. Assoc..

[37] Iovino F., Auriemma P.P., Dani L., Donnarumma G., Barbarisi A., Mallardo V., Calò F., Coppola N. (2017). Suture thread check test for detection of surgical site contamination: A prospective study. J. Surg. Res..

[38] McCaleb R., Warren R.H., Willis D., Maples H.D., Bai S., O’Brien C.E. (2016). Description of respiratory microbiology of children with long-term tracheostomies. Respir. Care.

[39] Rouzé A., Jaillette E., Poissy J., Préau S., Nseir S. (2017). Tracheal tube design and ventilator-associated pneumonia. Respir. Care.

